# A Brief Review of Personality in Marathon Runners: The Role of Sex, Age and Performance Level

**DOI:** 10.3390/sports6030099

**Published:** 2018-09-18

**Authors:** Pantelis T. Nikolaidis, Thomas Rosemann, Beat Knechtle

**Affiliations:** 1 Exercise Physiology Laboratory, 18450 Nikaia, Greece; pademil@hotmail.com; 2 Institute of Primary Care, University of Zurich, 9001 Zurich, Switzerland; thomas.rosemann@usz.ch

**Keywords:** aging, gender, master athletes, endurance, marathon, personality traits, psychology

## Abstract

The participation of recreational runners in sport events ranging from 5 km to ultra-endurance races have increased dramatically during the last decades and this phenomenon has attracted scientific interest. Most research has focused on the physiological characteristics of these runners and less in their psychological characteristics. Therefore, the aim of the present study was to review the existing knowledge with regards to the personality of recreational endurance runners and the role of sex, age and performance. It was concluded that limited information was available with regards to the personality of recreational marathon runners. So far, our knowledge on the personality of marathon runners relied on studies conducted a few decades ago, mostly on competitive marathon runners, highlighting the need for original research on recreational runners.

## 1. Introduction

The beneficial role of endurance exercise for health might explain partially the increase of participation in marathon races during the last decades [1]. With regards to sex differences in participation and race time, recent findings indicated that men were faster and older than women, whereas the participation of women increased disproportionately to that of men resulting in a decrease of the male-to-female ratio [2]. In addition, the number of master participants increased at a greater rate than their younger counterparts [3]. In elite master runners, the sex difference in performance in marathon was smaller than in shorter distances [4]. Most women marathon runners have been observed in the age group 30–34 years and most men in the age group 40–44 years [2]. Although studies on anthropometry, physiology and training characteristics have improved our understanding of the predictors of race time [5,6], psychological aspects of marathon runners, such as personality, have received less scientific attention so far. Nevertheless, personality, defined as the sum of characteristics that make a person unique [7], might be related with both participation and performance in marathon [8].

To the best of our knowledge, no review has been even conducted on the personality of marathon runners. Information from a review on this topic would be of great practical importance for strength and conditioning coaches, and sport psychologists working with marathon runners, especially considering the increased number of finishers and races during the last years. The recent trends in participation and performance were that relatively more women and older runners participated compared to the past, and overall race times became slower [2,9], highlighting the need for further research in the personality of recreational marathon runners. Therefore, the aim of the present study was to review previous original research on personality of marathoners focusing on differences in personality characteristics between women and men, age groups, performance levels and compared to other sports.

## 2. Psychological Profile

A general overview of the psychological profile of marathon runners has been derived from studies comparing them with general population. The cognitive anxiety, arousal, self-confidence, motivation and perception of physical state were the most representative variables of marathon runners psychological state [10]. The psychological profile of marathon runners has been characterized as “iceberg”, which indicated relatively low scores in tension, depression, anger, fatigue and confusion, and high score in vigour [11]. Moreover, it has been proposed that elite marathon runners were characterized by positive mental health and their ’effort sense’ [12]. Compared to the general population, middle-aged runners were more intelligent, imaginative, reserved, self-sufficient, sober, shy, and forthright [13]. In addition, marathon runners show higher levels of hardy personality (i.e., a group of characteristics related to personal perception of control, commitment and challenges) than general population [14]. The psychological characteristics of marathon runners vary with regards to the participation in a race. It has been observed that on the morning of the day of a race anxiety and hostility scores were higher than those on rest days, but depression and libido scores were unchanged [15].

Considering the limited existing data on marathon runners, the psychological profile of ultra-marathon and other sports was examined, too. For instance, a study on triathletes indicated low levels of anxiety and high levels of self-confidence [16]. It has been shown in Comrades Marathon (~90 km run) a strong identification with the activity, as well as social ethos and sub-culture influence in social identity [17]. In the TransEurope FootRace (4487 km), runners showed increased pain tolerance indicating that a low pain perception may predispose a person to become a long-distance runner [18]. In the Alaskan 100-mile run, findings suggested that those with an interest in exciting, new, and different activities might be well suited to participate in ultra-marathon [19]. In addition, a review of personality in sports showed that athletes were characterized by higher extraversion, emotional stability and responsibility compared to non-athletes [20]. These unique psychological characteristics of marathon runners should be regarded in the light of the centrally regulated and goal-directed exercise behaviour [21]. 

## 3. The Role of Sex

Several studies investigated the prevalence of menstrual irregularity and anorexia nervosa in women endurance and ultra-endurance runners. Menstrual irregularity has been experienced by ~40% of ultra-marathon runners during periods of intensive training due to emotional stresses of competitive ultra-marathon running, and menstrual patterns normalised once these stresses were removed [22]. In women distance runners, the incidence of abnormal eating attitudes symptomatic of anorexia nervosa was 14% and it was proposed that it was the better athletes who were more likely to exhibit the physical and psychological features of anorexia nervosa [23]. Women marathon runners did resemble anorexic patients in their scores, except that they did not appear to suffer as a result [24]. A research in high school girls showed prevalence of anorexia nervosa 1.5%, bulimia nervosa 2.0%, combination of anorexia nervosa and bulimia nervosa 0.3% and eating disorder not otherwise specified 12.9%, and found no relationship between the prevalence of eating disorders and the level of sport competition or time spent on physical activity. [25]. Female athletes, one in sports that provide an advantage to those with a thin body build and one in sports that demand a normal build, the first group even though they were thin, had greater weight and diet concerns, and were emotionally more labile and dissatisfied than the second group [26]. The development of eating disorders in women marathon runners might be associated with the brain reward mechanisms that generate “liking” and “wanting” for foods [27]. It was assumed that chronic food restriction might augment this “reward” mechanism [28]. Furthermore, eating disorders might be due to social-cultural and biological factors [29]. It should be highlighted that the causal-effect relationship between endurance sports and eating disorders might be bidirectional. For instance, it might be assumed that people with eating disorders would be engaged in endurance training to maintain a thin body build. Although these studies have enhanced our understanding of menstrual irregularity and anorexia nervosa in women, a lack of research in sex differences in personality was highlighted. Women may differ from men in commitment and negative addiction. For instance, it has been shown that women with small sport experience reported the “concerns of children and work” as reasons to train, in contrast to men who stated “to get away” or “get forgotten” [30]. On the other hand, a comparative study of marathon runners did not observe any sex difference in pre-competitive anxiety, cognitive or somatic [31]. 

## 4. The Role of Age

Runners participate in marathon races usually classified in 5-year age groups (e.g., 20–24, 25–29...). As the rate of participation and performance has been shown to vary by age group [2,4], potential differences in personality among age groups would be of great practical importance. One of the few longitudinal studies on marathon runners observed that most runners scored above the 85th percentile on boldness, warmth, conformity, sensitivity, dominance, and high drive with tension, and above the 93rd percentile for self-discipline and emotional stability [32]. The same study showed a distorted body image in half runners, whereas two developed anorexia nervosa, and another girl committed suicide suggesting a need for psychological screening for young children entering a strenuous training program. In another study, age groups of middle-aged runners (40–59 years) differed very little with regards to personality characteristics [13]. A comparative study in soccer suggested that top adult athletes were more emotionally stable and responsible than young players [33]. This finding was confirmed in soccer players between 14 and 21 years, where adult athletes obtained higher scores on the traits of emotional stability, openness to experience and responsibility than their younger counterparts [34]. In summary, few studies have been examined differences in personality among age groups of distance runners. Findings from studies in soccer might suggest that a similar trend would be expected in marathon runners, i.e. adults being more emotionally stable and responsible than their younger counterparts.

## 5. The Role of Performance Level

The increased rates of participation in marathon during the last decades were attributed mostly to the entry of relatively old age groups of recreational runners rather than to competitive runners [35]. Therefore, a major question was whether personality differed by performance level of marathon runners. To answer this question, two approaches were used; first, differences among marathon runners varying for race time were examined, and second, differences between marathon runners and other athletes were studied. Marathoners were observed to be introverts and judging types, whereas their differences from non-athletes were accentuated in sub-3h marathon runners [36]. A hardy personality can differentiate between marathon participants with the best and worst records [37]. They reported less depression, confusion, and more vigour than joggers, who in turn differed from non-exercisers [38]. They differed from joggers on reserved, less intelligent, serious, tough-minded, practical, forthright and self-assured [39]. Furthermore, a faster race time in marathon might be predicted by dispositional benign envy mediated via higher goal setting [40]. Thus, it might be supported that competitive marathon runners presented a distinct personality compared to recreational runners.

On the contrary, other studies did observe a similar personality between marathon runners and other athletes. For instance, no difference was found between women marathon and non-marathon runners in anxiety, self-esteem, family/spouse relationships and addictive behaviours [41]. Compared to cross-country skiers, marathon runners had similar scores on reserved, imaginative, self-sufficient, and independence; however, they appeared more intelligent and tough-minded [42]. A study of running marathon for first time showed differences in mood pre- and post-race indicating that participation in this race greatly effects mood, mainly in a more negative way than low to moderately intense exercise does [43]. 

Race time in marathon was related to and repression-sensitization (faster runners being more sensitized), but not to locus of control [8]. In addition, no difference was shown in marathon runners Type A and B (Jenkins Activity Survey) in finishing times or in subjective stress due to running in the marathon [44]. No difference in personality was found among ultra-marathoners, runners who had never raced any distance longer than 10 miles, and non-runners [45]. Compared to physically active men, competitive marathon runners were more morning-oriented indicating that individuals who train for and participate in recreational endurance sport races have an earlier chronotype than physically active but non-competitive males [46]. Women running 24 miles a week had healthier profiles on mood state than those running 52 or 15 miles a week [47]. The mood state of elite marathoners was similar as that of elite road-racing cyclists, oarsmen and wrestlers [48]. Athletes and non-athletes might adopt different emotion regulation strategies considering a model where strategies that act early in the emotion-generative process should have a different profile of consequences than strategies that act later on [49]. The role of emotion regulation for an optimal performance has been already highlighted in ultra-endurance cycling [50]. It has been acknowledged that what an athlete thinks about impacts on effort perception and performance [51].

## 6. Limitations, Strength and Practical Applications

The personality traits of marathon runners might have changed across time. The increased number of participants, especially of women and master athletes, in marathon races during the last decades resulted in a more recreational and less competitive profile of marathon runner nowadays compared to the past. Accordingly, the race time lowered, e.g., by 47 min from 1980 to 2014 in a USA marathon race and the age increased [52]. Thus, caution would be needed to compare personality traits of marathon runners between different chronological periods. Furthermore, most studies have used a cross-sectional design which did not allow establishing a causal relationship, e.g., whether a specific performance was achieved due to specific personality trait or vice versa, or whether low pain perception was due to the ability of running long distances or vice versa. Future studies should apply a longitudinal design, for instance, to study the effect of different training programs on personality traits. On the other hand, strength of the present study was its novelty as it was the first review to examine this topic. Considering the popularity of marathon among runners and researchers, the findings would be of both practical and theoretical application for practitioners working with runners and for scientists interesting in this topic, respectively. In addition, the organizers of races should consider sex, age and performance differences in personality traits in order to increase further the rates of participants.

## 7. Conclusions

In summary, limited information was available with regards to the personality of recreational marathon runners. So far, our knowledge on the personality of marathon runners relied on studies conducted a few decades ago, mostly on competitive marathon runners, highlighting the need for original research on recreational runners.
